# Coping in times of disruption and deprivation—Experiences of family members during COVID‐19 patients' critical illness: A qualitative study

**DOI:** 10.1002/nop2.1734

**Published:** 2023-04-02

**Authors:** Helene Berntzen, Ranveig Lind, Hanne Alfheim, Kirsti Tøien

**Affiliations:** ^1^ Division of Emergencies and Critical Care, Department of Postoperative and Intensive Care Nursing Oslo University Hospital Oslo Norway; ^2^ Department of Health and Care Sciences, Faculty of Health Sciences UiT The Arctic University of Norway Tromsø Norway; ^3^ Intensive Care Unit University Hospital of North Norway Tromsø Norway; ^4^ Faculty of Health VID Specialized University Oslo Norway; ^5^ Department of Anesthesia and Intensive Care, Vestre Viken Hospital Trust Baerum Hospital Baerum Norway; ^6^ Department of Research and Development, Division of Emergencies and Critical Care Oslo University Hospital Oslo Norway

**Keywords:** COVID‐19, family, family nursing, intensive care units, organizational policy, pandemics policy, visitors to patients

## Abstract

**Aim:**

To explore the experiences and needs of family members during the course of COVID‐19 critical illness from onset to rehabilitation.

**Design:**

An exploratory qualitative study.

**Methods:**

Twelve family members of surviving critically ill COVID‐19 patients and restricted from visiting the patients, were interviewed digitally. Reflexive thematic analysis was used.

**Results:**

Three themes were generated from the data; ‘Experiencing a double burden’, ‘Becoming an insignificant other’ and ‘Regaining significance’. Family members were often ill themselves, which represented an extra burden when the patient deteriorated. From admission, the family members became bystanders, deprived of most contact with the patients, as communication and information from the intensive care unit appeared unstructured and haphazard. However, when patients were discharged, great responsibility was placed on the family members.

## 1 INTRODUCTION

Family presence is crucial to critically ill patients in intensive care units (ICUs) and may improve outcomes for these patients (Goldfarb et al., 2017). International position statements and guidelines clearly highlight the paramount position of the family in critical care (Davidson et al., 2017; Mitchell et al., 2015). Despite these recommendations, ICU visiting policies around the world vary from flexible to restricted (Cappellini et al., 2014). During the COVID‐19 pandemic, most ICUs were closed to visitors, leaving ICU patients without close contact with their family members (Jaswaney et al., 2022; Jensen et al., 2022; Rose et al., 2021).

Critical illness and admission to an ICU have a major impact on patients' family members (Shaffer et al., 2016) and they suffer from psychological distress both during and after the patient's ICU stay (Alfheim et al., 2019). Post Intensive Care Syndrome‐Family (PICS‐F), a cluster of symptoms including anxiety, depression, post‐traumatic stress symptoms and complicated grief, describes this distress in family members of critically ill patients (Davidson et al., 2012). Family members have reported not being allowed to visit ICU patients due to COVID‐19 restrictions as a terrible, traumatic burden. They have expressed frustration, anger (Digby et al., 2022) and fear of what could happen to their loved ones (Bartoli et al., 2021). Family members' presence in the ICU is crucial in providing and receiving care. A comparison of restricted and open visiting hours in ICUs pre‐pandemic (Rosa et al., 2019) revealed that family members in units with limited visiting hours had significantly more anxiety and depression than those in units with open visiting policies.

Visiting restrictions deprived family members of face‐to‐face contact with their loved ones and ICU staff, thereby hampering communication and leading to psychological suffering (Moss et al., 2021; Zante et al., 2021). Therefore, many ICUs established telehealth solutions to enhance communication with family members outside the hospital (Kebapcı & Türkmen, 2021; Rose et al., 2021).

Family‐centred care highlights the importance of involving the family during the patient's stay in the ICU and the responsibility of clinicians to provide emotional support to the family (Davidson et al., 2017). In this approach, one central element is physical presence of family members, facilitated by ICU–clinicians (Al‐Mutair et al., 2013; Olding et al., 2016). The importance of nurses promoting engagement with families is presented in a theory by McAndrew and colleagues (McAndrew et al., 2020). They describe how ICU nurses are surrounded by factors that facilitate or disrupt this engagement. The main aim of the theory was to improve families' opportunities to engage in patient care, and the core proposition is that this enhances the outcome for both patient and family members (McAndrew et al., 2020).

In addition to families being deprived of contact with the ICU, the closing down of society across the world reduced social interaction and increased social isolation (Vacher et al., 2022). Family members' lack of support from their usual social network might have had a negative effect, since a high level of social support has been associated with lower levels of post‐traumatic stress symptoms, anxiety and depression in family members of patients during the ICU stay (Azoulay et al., 2022).

Visiting restrictions were justified by the need to prevent the spread of COVID‐19 and the anticipated shortage of ICU beds and personal protective equipment. The pandemic visiting restrictions have been described as ‘an outbreak of restrictive ICU visiting policies’, which was not well justified (Dos Santos & Nassar, 2022), questioning the rationale for protracted restrictions.

Research on the consequences of visiting restrictions during COVID‐19 has focused on both healthcare workers, patients and family members (Moss et al., 2021). Several quantitative studies have shown that family members of COVID‐19 ICU patients suffer from psychological distress (Amass et al., 2022; Azoulay et al., 2022; Greenberg et al., 2021; Kosovali et al., 2021; van Veenendaal et al., 2021; Vincent et al., 2021; Zante et al., 2021). Five qualitative studies have explored the specific experiences of family members of surviving COVID‐19 ICU patients (Bartoli et al., 2021; Bernild et al., 2021; Chen et al., 2021; Greenberg, Basapur, Quinn, Bulger, Schwartz, Oh, Ritz, et al., 2022; Klop et al., 2021). However, only two of them focused on family experiences throughout the whole trajectory of the COVID‐19 illness (Bartoli et al., 2021; Chen et al., 2021). Knowledge about the whole trajectory is important to understand the complexity of family members' experiences during the patient's critical illness and visiting restrictions. By increasing our knowledge on the consequences of excluding family members from ICUs may possibly prepare us for future resembling situations.

## 2 METHOD

### 2.1 Aim

The aim of the study was to explore the experiences and needs of family members during the course of COVID‐19 critical illness, from onset to rehabilitation.

The research questions were as follows:

- How did the family members experience the visiting restrictions during the patient's critical illness?
- How did the family members experience the time before admission and after discharge of their ill relative?

### 2.2 Design

In this qualitative exploratory study, we interviewed adult close family members of COVID‐19 ICU patients during the first and second wave of the pandemic. The study is reported according to the Consolidated Criteria for Reporting Qualitative Research (COREQ) (Tong et al., 2007).

### 2.3 Participants and setting

Purposive sampling and snowball recruitment were used (Patton, 2015). Inclusion criteria were being an adult (≥18 years) close family member of a COVID‐19 patient in need of critical care and hospitalized in one local and one university hospital in Norway and being able to understand a Scandinavian language. Close family members are in the Norwegian setting defined as whoever the patient sees as family, but all participants in this study were also legal family members. After the first pandemic wave or peak of hospital admissions, we included family members retrospectively by identifying eligible participants through patients in the local COVID‐19 registry. We sent a letter asking all discharged patients with an ICU—stay of more than 48 h to invite their closest family member to participate in the study by responding to the author, a female critical care nurse researcher (KT). In the second wave, we included family members prospectively through nurses responsible for patients. They asked the family member to allow the author (KT) to contact them about the study, and if consenting they were informed and asked to participate.

Cohorts of COVID‐19 patients in need of critical care were organized and staffed across different ICUs with both critical care nurses and nurses without ICU training. Norwegian critical care nurses work in accordance with international recommendations for family care, which consider family members as valuable resources for the patient and the ICU team and recipients of care (Institute for Patient‐ and Family‐Centered Care, 2022; Davidson et al., 2017; Mitchell & Denise., 2015). Before COVID‐19, guidelines in the ICUs studied allowed family members to visit their close relatives without restrictions, but with recommendations for avoiding doctor's rounds and patient resting time. From the onset of COVID‐19 in March 2020, visitors' access to ICUs was prohibited, with a few exceptions for patients at the end‐of‐life.

**TABLE 1:** Interview guide.

	Overarching questions	Support questions
1	Suddenly being a family member of a COVID‐19 ill loved one in the ICU?	How did you feel when it happened? Did anyone else in your close family support you? Were you able to sleep?
2	Were you at all allowed to come in to visit your loved one in the intensive care unit?	If not, what kind of contact did you have with doctors and nurses? Did you use telephone/video? What kind of feelings and thoughts did you have about not being allowed to visit?
3	What did the doctors and nurses talk about with you?	Did you get the information you needed? Did you feel that they cared for you? Were you comforted?
4	Experience of hope	What gave you hope during the time your loved one was in the intensive care unit?
5	Coming home	How did you feel when your loved one came home? Please describe emotions, practical challenges, support/follow‐up
6	As a family member, did you receive any follow‐up afterwards?	In which way? What has it meant to you?

### 2.4 Data collection

Data were collected using individual semi‐structured interviews with one close relative of 12 COVID‐19 patients admitted to an ICU. Patient data were retrieved from the local COVID‐19 registry: gender, age, length of ICU stay, length of hospital stay, ventilator treatment and severity of illness.

All interviews were conducted virtually from December 2020 to April 2021 by one of the researchers (KT) using a secure hospital platform. The interviews lasted approximately 1 h. The participants were first invited to speak freely about what happened during the days and hours immediately preceding ICU admission of their close family member. An interview guide was used based on previous research and developed by the researchers after discussions with two user representatives (one former ICU relative from the first COVID‐19 wave and one from the pre‐pandemic period). The guide focused on experiences of being a family member of a COVID‐19 ICU patient (Table 1). The criteria of study aim, sample specificity, quality of dialogue and analysis strategy, representing information power (Malterud et al., 2016) were considered obtained after 12 interviews, all comprising rich data. The interviews were audio‐recorded, transcribed verbatim and stored and analysed in a secure university and hospital platform, TSD.

### 2.5 Analysis

We analysed data using a reflexive thematic approach (Braun & Clarke, 2021) and used NVivo software (QSR International) for data management. Two researchers (HB and RL) worked independently and together throughout the analysis. Themes were generated in a reflective approach through six phases. The first phase was familiarization with the data, after which the interview text was coded across cases in a subjective and reflexive process. The two researchers met several times in the process of generating themes and then reviewed and refined them in additional discussions. Preliminary themes were presented and discussed with the rest of the research team (KT and HBA) and in a meeting with the user representatives. After this, HB and RL defined and named the final themes.

Trustworthiness in this study is described by Lincoln and Guba's framework (Lincoln & Guba, 1985; Shenton, 2004). Credibility was ensured by applying a well‐recognized research method and analytic approach, through keeping a reflective commentary throughout the analysis and using thick descriptions and quotations from the interviews. In addition, two former family members of ICU patients, one of these in isolation, participated during the research process. All analytical steps were performed by at least two researchers to prevent bias, ensure different perspectives and to enhance confirmability. Dependability was sought by transparency through detailed descriptions of the research process allowing the reader to assess the research practice. Transferability to other contexts was sought through descriptions of the phenomenon under study, the setting and participants.

### 2.6 Ethical considerations

The study was conducted according to the Declaration of Helsinki (Rohrich, 2013). Family members received written and oral information about the study and returned written consent to participate by post. Written consent was also obtained from the former patients in order to use demographic and clinical information from the COVID‐19 registry. The hospital Data Protection Officer approved the study (IRB 2020/21937). The Regional Committee for Medical and Health Research Ethics waived to process the application, since no patients participated in the study. Since the interviews might have activated traumatic memories, the family members were invited to call the interviewer (KT) if they experienced difficult feelings after the interview. However, no one made use of this offer.

## 3 RESULTS

The overarching theme ‘coping in times of disruption and deprivation’ represents the essence of the participating family members' experiences of being a close relative of a critically ill COVID‐19 patient in the ICU. The three themes ‘experiencing a double burden’, ‘becoming an insignificant other’ and ‘regaining significance’, together with nine subthemes, underpinned the overarching theme (Table 3). The experiences of the relatives in this study involved three major phases. Firstly, the frightening onset of the COVID‐19 infection, secondly, the disruption caused by hospital admission with visiting restrictions and thirdly, the return home of a family member still in need of extensive care.

The results are presented through descriptions of what the relatives were deprived of as a result of the unique situation and of how they coped with this, reflecting the overarching theme; ‘coping in times of disruption and deprivation’.

**TABLE 2:** Overview of the overarching theme, themes and subthemes.

Overarching theme: Coping in times of disruption and deprivation		
Themes		
Experiencing a double burden	Becoming an insignificant other	Regaining significance
Subthemes		
Being ill and insecure Feeling responsible and self‐reproachful	Losing sight of the loved one Yearning for information Protecting others and oneself Seeking control and predictability Negotiating the unreasonable situation	Being unprepared and unsupported Compensating and contributing

### 3.1 Participant characteristics

Twelve family members were interviewed, two men and ten women (Table 2). Seven were partners, four were children and one was a mother. The participants were aged between 27 and 72 years. Seven were relatives during the first phase of the pandemic, in spring 2020 and five were relatives in the second phase. None of the participants had been visiting physically in the ICU, but a few who were relatives during the second wave had been visiting in the medical or rehabilitation ward following the ICU stay.

**TABLE 3:** Characteristics of family members, patients and their relations.

Participant	Gender, Male/female	Age	Relation to patient	Pandemic phase on admission, month/year	Patient length of stay in ICU	Ventilator treatment, days
1	F	50	Wife	03/20	4	0
2	F	72	Partner	04/20	5	0
3	F	52	Daughter	04/20	2	0
4	F	63	Mother	03/20	3	0
5	F	55	Partner	03/20	5	0
6	M	44	Son	04/20	20	16
7	F	55	Wife	03/20	10	9
8	F	67	Wife	12/20	14	0
9	M	27	Son	01/21	16	12
10	F	49	Wife	01/21	20	0
11	F	46	Daughter	01/21	31	28
12	F	44	Wife	02/21	8	0

### 3.2 Experiencing a double burden

#### 3.2.1 Being ill and insecure

A main finding was the double burden resulting from being a concerned caregiver of a relative in deteriorating health, while also experiencing threats to one's own health. The latter caused profound insecurity about being infected with COVID‐19 and the possible consequences. The extra burden experienced by several relatives who were actually ill themselves was evident, whether they were ill at admission, during the hospital stay or when their loved ones returned home. Along with their concern for their hospitalized relative, they all struggled with the concurrent general insecurity of the pandemic situation, including a lack of established systems for COVID testing and counselling on isolation and quarantine.

He said he tested positive for COVID and then I got a call telling me to go into quarantine. I said yes, but asked if I could please be tested…No, only if I had symptoms… That was probably the most distressing part – having to go into quarantine for the second time without a test. (Partner, 72')

One wife had already been quarantined for 2 weeks with her two children due to her husband's illness and hospital admission. He was discharged home but deteriorated quickly and was readmitted within 2 days. It was quite unclear what rules applied to the family in this case. Besides being extremely frightened about their father and husband, they were beginning to suffer from the social isolation.

I was very unsure about things! Were we in isolation? And a lot of different things were said… – and from the infection control office we never heard a thing. (Wife, 50')

#### 3.2.2 Feeling responsible and self‐reproachful

Family members were key persons in making decisions regarding their ill relative's deteriorating health status. Emergency telephone lines were busy and people were generally discouraged from seeing their doctor or visiting emergency outpatient clinics in order not to spread the virus. This resulted in a lack of decisional support in relation to the admission, which put a strain on the relatives.

….and the worst part was actually the time before he was admitted, no one from healthcare came to see him… there was nothing…and it took far too long before he was taken seriously. If I'd known, I'd have been pushier, been tougher in a way and had him admitted… (Partner, 55')

Not living with the ill person was no less demanding for the relatives. The mother of a young boy living in another part of the country said:

I called the emergency phone where he lives three times. The third time, he got help and the ambulance came for him…His temperature was almost 41 when he got to the hospital, and that's a lot. (Mother, 63')

Several of the relatives described a cognitive influence of the virus infection that sometimes made the patients not realize how ill they were. In these cases, the relatives had to be decisive and even override the patient's wishes about contacting the healthcare system.

He acted totally strange… finally I thought: this isn't working, so I just called the ambulance – and he got furious when I told him they were on their way …or actually right outside… He walked down the stairs like a sulky kid and … I guess it was the last I saw of him. (Wife, 55')

The nature of the disease also made some participants trivialize their family member's symptoms and one family was devastated when the husband and father was acutely admitted to intensive care. Being responsible for taking action—or not—could trigger a feeling of guilt in the relatives in either situation.

### 3.3 Becoming an insignificant other

Another finding was the deprivation of normalcy in the role one is expected to ‘play’ as a relative of someone critically ill. Hospital visiting restrictions deprived relatives of the possibility to support their ill family member during the illness and of receiving support from healthcare personnel.

#### 3.3.1 Losing sight of the loved one

Seeing their loved one off, either in the ambulance or through the hospital doors was upsetting to many, given the insecurity of the situation.

I handed him over to those infection control people and knew this was what he would be dealing with from now on, but not for how long. I didn't feel so emotional about myself – that it was hard on me, but I felt so bad for him and the children. I don't really think there was any room in me for more emotions. And of course, I was awfully scared that he would die. (Wife, 50')

Almost none of the family members in this study were allowed to visit their ill relative during the hospital stay and none during the ICU stay. From the normal position of being a significant other for the patient, the family members became distant and insignificant as caregivers.

…it was awful, sad you know, not to be allowed to see your son when he was so ill. And we didn't know if he would survive. We wanted to see him before he died. Those were the kind of thoughts running through my head. (Mother, 63')

Facing the life‐threatening situation of their family member, some found it particularly distressing not to be able to see their loved one to be reconciled or make amends.

We had been teasing him, you know (because of his whimpering), and now we've all got a guilty conscience about it (…). Both the boys went through a life crisis. And you know, let´s say we could have seen him in the hospital, then we could have told him: “We didn't mean to tease you, and we can see now you're very ill, but at that time, we couldn't”. (Wife, 50')

Most relatives expressed gratitude and trust in the hospital and the healthcare staff. However, not being allowed to visit the patient, relatives could not see the condition of the patient for themselves. Being unable to be there to care and to see for herself, the wife of one patient said she was hoping one of the nurses would fall a little in love with her husband to ensure the provision of good care. Small glimpses of engagement and personalized care provided comfort:

He (the doctor) talked about things that ‘Peter’ had told him, from our life together, and then I felt “Oh, they've got to know him, they care about him…” (…) otherwise it's just kind of automatic, you know, “He's like this and like that…” and I don't even know if they've been talking to him… (Wife, 55')

A major consequence of the visiting restrictions was how they affected the information provided to the relatives.

#### 3.3.2 Yearning for information

Following admission of their loved ones to the hospital, the relatives' quest for information started. Deprived of the possibility of seeing their loved one in the ICU, they were yearning to know how he or she was doing. Information was in some cases provided daily, even several times a day. But more often it was provided in an inconsistent or arbitrary manner, regarding how it was obtained, who would provide it, how often, at what time and in what way. It appeared to depend largely on the individual nurse or doctor in charge of the patient on a particular shift. However, it remained unclear who was mainly responsible for providing the information and one relative stated:

…well, the nurses sort of hide behind the responsibility of the doctor, and they're afraid of saying too much …of saying the wrong things (…). It was so nice when the nurse explained to me: “Now he's breathing so well, his face looks good” …because I couldn't see him, you know (…), but I wish she could have told me some more about what they obviously know… (Wife, 55')

A few of the relatives felt well informed and experienced a certain routine, whether they spoke regularly to their relative on the phone, called the hospital themselves or were contacted by the clinicians. Some mentioned names of clinicians who had been very important to them.

…there was this constant message: “you may call at any time” and “you should call, and not think about us being busy, because we're here for you as well (…)” So I felt that I could have called in the middle of the night, if that was what I needed. (Partner, 72')

However, many relatives felt deprived of the information they needed to cope with the situation. In most cases, the nurses were caring when they spoke to them, but they hardly spoke to the same nurse or doctor more than once or twice and generally not often enough. Their comments revealed a frustrating lack of structure or system of the information provided by the ICU to the relatives at home.

I would have preferred more regular calls, that they set aside some time when it's not possible to visit and be with the patient and it's all closed… I had a great need (for information) all the time and I wanted a better system – optimally, if I'd only known: okay, ten minutes before change of shift they'll call me. (Wife, 55')

Some felt rejected when trying to contact the hospital and some were explicitly told not to phone, but to wait to be contacted by the hospital. The days ‘consisted of waiting’, one relative said. Telehealth communication was not offered, but FaceTime was used on a few occasions when the patient was able to use his or her own telephone. Seeing their loved one was highly appreciated. It provided a more complete picture of the situation and could also facilitate caring for relatives at home through a more personal encounter, involving the patient, the relative and the nurse.

We had “Face” on the phone, and I saw him lying there with the mask and loads of equipment and things, and to be able to talk to him and read what was possible from his face …it was enough for me – and sometimes the nurses kind of stuck their face into the camera and said hello. I remember saying one day that I was going for a walk, and she went: “Oh I wish I could join you, but I'll look after ‘John’ instead”. That was really nice. (Partner, 72')

#### 3.3.3 Protecting others and oneself

Facing the difficult situation of neither having access to the critically ill family member nor being provided sufficient information to relieve their concerns, the family members used certain strategies to protect themselves from general information about the pandemic. They mentioned in particular the dramatic, frightening scenes on the TV news from COVID ICUs in Italy. Lacking specific information on their own relative's condition and environment, these images became a replacement that was easily applied to their loved ones, making them terrified. The relatives described how they avoided news broadcasts and how they sought distractions from the COVID‐19 situation.

We couldn't stand the news at all. So I've literally been sitting down with my boys watching Ex on the Beach and Paradise Hotel because it was all we could bear to watch (…) I'd never have thought I'd watch those things but seeing young people having sex and getting drunk on TV… – it was actually quite pleasant because it kept my mind occupied. (Wife, 50')

The relatives acted protectively towards other family members, especially young children, by providing selective information in small doses and by standing strong. One young son who took on responsibility for his mother and siblings when his father was admitted said:

I was much more distressed than I showed, at least the first weeks. I thought of it constantly and drove around a bit just to sit in the car and get rid of some thoughts and scream (…). It was really tough. (Son, 27')

#### 3.3.4 Seeking control and predictability

Worrying about their family member's condition, relatives were left to their own existential reflections on the perceived life‐threatening situation. Several described how they went on the offensive and made plans for the funeral, started planning for a future on their own, and worried about if they would be able to keep their house or how they would cope as a single parent.

You get to be a bit practical too. I checked our insurance policies…you know, will I have enough on my own if he dies? (Wife, 50')

Another way to meet the demanding situation was by attempting to create a more predictable daily life. Predictability was sought through daily routines of physical activity, regular telephone calls with other family members, and although rare, scheduled contact with the hospital.

#### 3.3.5 Negotiating the unreasonable situation

Despite the horrific situation of not knowing, expecting the worst, not being able to visit and see for themselves, and the general lack of contact, information and support from the ICU, there were few complaints. Some naturally wished the information had been better, but a general impression was the relatives' admirable acceptance of the circumstances. In different ways, they adapted to the situation and how it affected them. They said there was no other way that thinking more of themselves in the situation would be unreasonable and that they were grateful that the healthcare workers focused on the patients and not on their family.

I'm so glad the focus was on helping Mum – and it seems unrealistic and demanding to expect them to attend to my needs. (Daughter, 52')

Another way of adapting was to declare ‘no news is good news’ when they lacked information, and to claim it would probably have been more traumatic to see their relative in the ICU than not, when they were unable to visit.

### 3.4 Regaining significance

The last theme covers the return home of the patients and the following period. In this phase, the caregivers regained their position as statistically significant others. However, coping had taken its toll as many caregivers were still weak following their own COVID illness, were already overburdened by their home situation and had little support from outside.

#### 3.4.1 Being unprepared and unsupported

Upon returning home, the patients were still in need of extensive care; a situation for which the relatives were unprepared and hence concerned. There were no discharge interviews, written information or preparations and there was no follow‐up care in the immediate phase following discharge.

So I picked him up in the car. There he was, outside the hospital and there was nothing, you know… Later on, I thought they should have had a discharge conversation with someone… (Partner, 55')

One wife talked about how her husband returned home with a ‘pile’ of anticoagulant syringes but he claimed he was only going to use three of them. Several missed information about possible reactions to the hospital stay and about how to deal with these. Sometimes conflicts arose:

If someone could have talked with both of us about common reactions and told him that his family has been through something different from him – just as hard, only different. I believe that kind of conversation could have made things easier for us later. (Wife, 55')

#### 3.4.2 Compensating and contributing

At discharge, some were still defined as patients with great personal care needs, with physical and cognitive impairments and with reactions to their ICU stay. Relatives described how they coped in supporting the physical and psychological needs of the returning patient. One wife told how she spread chairs all around to ensure that her husband always had somewhere to sit down to regain his breath. The relatives returned to a position of being statistically significant others as their knowledge and understanding of their loved one was crucial in this situation.

…and then he asks me: “Do I need a password to sleep?” (…) Sleep had been a serious issue, so he wasn't quite all there, you could say. But then I got him to start drawing. He's really good at drawing and painting, but he has not done it for years, so I just made him draw, because then you focus more on reality. (Wife, 67')

Some also involved friends or family in the support. One wife told how a physiotherapist friend came to their garden and from a distance instructed her husband how to exercise.

This coping related mostly to relatives living with the patients, but those who witnessed the return home from a distance were highly concerned and regretted that they could not be on hand to help.

He was still ill when he got home, had breathing problems and so on. And he said he was scared to go to sleep… that he wouldn't wake up again (…) he has those thoughts… waking up from nightmares at night and then he calls home …he's done that several times. (Mother, 63')

There was generally little follow‐up care, although some patients had contact with their general practitioner and some in the second phase were offered follow‐up consultations at a clinic.

## 4 DISCUSSION

The main findings in this study reflect the overarching theme ‘Coping in times of disruption and deprivation’. This relates to the disruption of family bonds or processes caused by the patients' ICU admission and is reinforced by the visiting restrictions. Furthermore, the findings show how family members experienced and coped with the deprivation of support and information prior to, during and following the hospital stay. The status of the family members appeared to alternate between statistically significant and insignificant during the patients' illness trajectory. The significance resulted from the great responsibility and even burden placed on family members before admission and following discharge. The insignificance, however, resulted from the general lockdown of society and visiting restrictions in hospitals, furthermore the unsatisfactory communication with healthcare workers.

According to Ho (2008), critical illness brings moral significance to people's intertwined existence, with a human obligation to take part in each other's lives (Ho, 2008). Being involved in the patient's illness as the closest family member is hence fundamental and has been described as ‘twosomeness sharing one life’ (Vester et al., 2020). Being separated as a couple due to critical illness can threaten this unique relationship and make the ICU experience very stressful. Adding to this burden in our study was the general lockdown of society that deprived many of the participants of support from people around them, often leaving them alone and insecure. This may indicate that family care in general should include a structured examination of family members' experiences.

Being able to visit and engage in patient care are core family needs. This also includes receiving and giving information (McAndrew et al., 2020; Olding et al., 2016). In line with this, family members are found to be ‘a connecting link’ within the ICU team, creating continuity and good information flow about the patient (Nygaard et al., 2022). In contrast to our participants' significance prior to and after the hospital stay, the general visiting ban compromised these fundamental elements of family involvement in the ICU. Family members participating in this study worried about whether the nurses had enough information and knowledge about the patient to compensate for their important role as family members. This is in line with previous research highlighting how nurses experience patients to be objectified due to the lack of input from family members (Andersson et al., 2022a, 2022b). Another study found that some ICU nurses missed the family presence in the process of getting to know the patient (Stenman et al., 2022). This elucidates the reciprocity in nurses' and family members' needs when caring for and caring about the critically ill patient.

In our study, having needs met and being supported as family members was inevitably compromised because of the visiting ban. A few were supported by telephone calls from what appeared to be individual initiatives from community health care, which was highly valued. This is in line with findings from an ICU setting where a structured family support team made daily calls to family members (Klop et al., 2021). In our study, no such structured interventions were described. Rather, our participants adapted to the very difficult situation of being restricted from visiting the ICU and there was a surprising lack of dissatisfaction and complaints. All participants expressed trust in the government decisions to close down society including visits to hospitals, to prevent the spread of COVID‐19. In contrast to this, (Digby et al., 2022), describe how family members in their study were frustrated and angry despite having permission to visit patients for 1 h each day. The acceptance of visiting restrictions in our study might reflect an overall loyalty towards governmental regulations (Jensen et al., 2022). Despite showing admirable compliance with this extraordinary situation, the family members found it hard to accept the near cessation of contact and communication with the patient and healthcare personnel. Being forced to accept visiting restrictions that largely contradicts their own conviction of what is best for the patient may add extra burden to the family member. The compliance shown might illustrate one aspect of the negotiation used by the family members to cope with the situation. To reveal family members' suffering, this may be important for healthcare personnel to investigate.

A major finding in our study was that communication between family members and clinicians appeared highly variable, unsystematic and with unclear responsibilities between nurses and doctors regarding family care. What appeared to be lacking was the care, communication and support usually provided in contact with nurses during an ICU visit, described by McAndrew et al. (2020) as the core element of nurse‐promoted family care. The yearning for more structured communication corresponds to what is reported in other pandemic studies (Bartoli et al., 2021; Bernild et al., 2021; Chen et al., 2021). A recent pre‐pandemic qualitative study showed how critical care nurses take overall responsibility for family members during their visits to the ICU, providing them with comfort, support and education about the patients' situation. The often busy doctors leaned on nurses' family care and waited to be summoned when the nurses need support, primarily for information tasks (Nygaard et al., 2022). In line with our findings, a US survey showed that doctors took on considerable responsibility for ICU patients' family members during the pandemic, which was found both time‐consuming and distressing during their clinical work (Wendlandt et al., 2022). Our findings of unclear team roles along with the many challenging conditions of the pandemic, may have contributed to the nurses' inability to fulfil the needs of the family members.

Only a few of our participants were offered virtual contact with the patient or a clinician, whereas many ICUs worldwide had organized telehealth solutions to establish virtual communication with family members (Kebapcı & Türkmen, 2021; Rose et al., 2021; Sasangohar et al., 2021), The very few virtual visits offered to the family members in our study were highly appreciated. The visual sense helped these family members to understand the patient's situation and to reduce their level of anxiety. They also said that they could support the patient by commenting on improvements in the patient's condition. Virtual solutions have been widely supported during visiting restrictions. It has been regarded as essential to support critically ill COVID‐19 patients and their families (Jeitziner et al., 2022) and has shown to reduce the prevalence and magnitude of severe distress in family members (Rose et al., 2021). Yet one study reported that family members found virtual visits to be of no help (Chen et al., 2021). This is in line with findings in the present study where two of our participants claimed they were probably better off not having seen the patient, neither live nor on video. These kinds of adaptations were interpreted as means to cope with the stressful situation of being restricted from visiting patients. In a randomized controlled trial, satisfaction and emotional well‐being improved in family members who received daily written updates on the patient's status to support the usual communication with the medical team (Greenberg, Basapur, Quinn, Bulger, Schwartz, Oh, Shah, & Glover, 2022). Such creative interventions might represent feasible efforts to meet the needs of family members during visiting restrictions and as a means to provide more systematic information when technical resources are scarce or guidelines are lacking. Other ICUs reported following checklists to ensure that all necessary topics were covered during conversations with relatives (Negro et al., 2020).

Our participants' experiences appear to be existentially horrendous. Following the visiting restrictions of ICUs, little is known about the long‐term consequences for ICU patients' family members. However, Greenberg et al. (2021) found that family members suffered more from depression and post‐ traumatic stress symptoms during the pandemic than family members before its onset (Greenberg et al., 2021). According to Davidson et al. (2012), being excluded and unable to fulfil obligations as the closest family member heightens the risk for symptoms such as anxiety, stress and depression. Prevention of PICS‐F has focused on family presence in the ICU, dedicated communication and the provision of emotional and social support to prepare the family for patient discharge (Inoue et al., 2019). As all the preventative measures were hampered by COVID‐19, there is reason to believe that our participants run a risk of developing PICS‐F. Moreover, the reported distress continued and was still present when interviews were arranged several months after the ICU stay.

Although visiting restrictions were seen in our study as a necessary means to reduce the spread of COVID‐19, routines to compensate for family presence, care and involvement appeared to be limited, compromising the care of family members. McAndrew et al. (2020) in their theory point to facilitators and disruptors for nurse‐promoted family engagement in the ICU. Facilitators of family adaptation include organizational responsiveness, unit support and ICU nursing culture. Disruptors include system barriers, ethical conflicts, family distress and family exclusion.

Organizational responsiveness comprises a culture that supports nurses to deliver care to families. The COVID‐19 situation was exceptional and demanding for all levels in the healthcare system. Family involvement apparently required a strong organizational policy and could not rely merely on nurses' individual initiatives, not even on local unit support as these were under much pressure. In our study, the organization or management appeared to fail in supporting clinicians to maintain good information and communication practices. Although critical care nurses' culture for the provision of family care can be an important counterweight to societal and organizational regulations and disruptors like visiting restrictions in the ICU, it is also necessary to address system barriers (McAndrew et al., 2020). The random use of FaceTime conversations found in our study was clearly not part of an established organizational procedure. Rather, it may indicate that nurses acted individually to counteract an ethical conflict disruptor (McAndrew et al., 2020) to maintain a minimum of family involvement. Jeitziner et al. (2022) in their Delphi study conclude that hospital or ward management should provide safe technical equipment for ICU care and communication. This will include the provision of safe systems and equipment to facilitate telehealth communication. Our findings may further indicate that the ICU units in this study, and other Nordic ICUs (Jensen et al., 2022), possessed a weak culture of patient‐ and family‐centred care. In a nursing culture that lacks an explicit family care policy, family members may be left with highly variable and unsystematic care.

### 4.1 Methodological considerations

This study has limitations. The sample was small and only family members of patients surviving the ICU stay were included. Family members of patients who died may have had other experiences. Furthermore, the interviews were conducted virtually, creating a distance that may have hindered the building of rapport between the participant and the interviewer. On the other hand, data from all interviews were rich, possibly indicating that being in their own home when speaking about personal matters may have increased the confidence and openness of the participants.

The interviewer was a part‐time employee at the unit where the patients were admitted and the participating family members were aware of this. This close position may have affected the participants' willingness to share experiences reflecting negatively on the unit or the staff and may also have influenced the analytical approach. Two other researchers, therefore, took the main responsibility of performing the qualitative analysis, yet included the interviewer in discussions on how to understand the data.

The interviews were performed several months after the patient's discharge from the ICU. Experiences during and following the ICU stay might have been processed and memories could have faded.

## 5 CONCLUSION

The visiting restrictions clearly hampered the mutually beneficial partnership, which normally applies during family‐centred care in the ICU. Family members were forced to be bystanders on a critical illness journey where they were deprived of most contact with the patients, and where communication and information from the ICU appeared unstructured and haphazard. The visiting restrictions prevented them from fulfilling their expected role as a family member and they thus became insignificant others. In addition to having minimal contact with the ICU, they were deprived of contact with their own social network. Attempting to cope in this very difficult situation, our participants protected themselves through avoidance, sought control and negotiated the unreasonable situation.

The distress expressed by participants in this study indicates that the healthcare system was not sufficiently prepared to support family members. Many facilitators of care regarding family engagement appeared to be lacking, while disruptors were present, explaining to an extent the major change and step backward seen in care for family members and subsequently patients. Our findings suggest an urgent need to establish routines and competencies to prepare for future situations involving visiting restrictions or bans.

Our findings on how family members experienced being excluded from the ICU may be transferable to other situations where families are unable to visit and see critically ill relatives due to isolation, long distances, their own illness or other reasons. Family‐centred care models involving openness and flexibility towards families are crucial and merit the attention of organizations and hospital units to ensure a more resilient practice when the physical presence of family members is impossible.

## 6 RELEVANCE TO CLINICAL PRACTICE

The acknowledgement of close family members as intertwined with the critically ill patient and in need of information and support, is pivotal in times of visiting restrictions in order to help them to cope with anxiety and distress, as shown in our study. To compensate for the lack of physical presence, a structured practice should be established to enhance contact and communication between family members and clinicians and patients when possible. Maintaining contact, such as through virtual visits, may support family members and guide the ICU team to provide personalized care to the patient as a unique human being ‘belonging to someone outside the ICU’.

## AUTHOR CONTRIBUTIONS

KT, RL and HA involved in conceptualization. KT collected the data, RL, HB and KT formally analysed the data. All authors involved in the methodology, wrote the original draft, reviewed and edited the manuscript.

## FUNDING INFORMATION

Departmental funding only.

## CONFLICT OF INTEREST STATEMENT

All authors declare no conflict of interest.

## Data Availability

The data are not publicly available due to privacy or ethical restrictions.
